# The implications of community responses to intimate partner violence in Rwanda

**DOI:** 10.1371/journal.pone.0196584

**Published:** 2018-05-02

**Authors:** Jenevieve Mannell, Iran Seyed-Raeisy, Rochelle Burgess, Catherine Campbell

**Affiliations:** 1 Institute for Global Health, University College London, London, United Kingdom; 2 Independent researcher, Utrecht, Netherlands; 3 Firoz Lalji Centre for Africa, London School of Economics and Political Science, London, United Kingdom; 4 Department of Psychological and Behavioural Sciences, London School of Economics and Political Science, London, United Kingdom; University of Westminster, UNITED KINGDOM

## Abstract

Intimate partner violence (IPV) has significant impacts on mental health. Community-focused interventions have shown promising results for addressing IPV in low-income countries, however, little is known about the implications of these interventions for women’s mental wellbeing. This paper analyses data from a community-focused policy intervention in Rwanda collected in 2013–14, including focus group discussions and in-depth interviews with community members (n = 59). Our findings point to three ways in which these community members responded to IPV: (1) reconciling couples experiencing violence, (2) engaging community support through raising cases of IPV during community discussions, (3) navigating resources for women experiencing IPV, including police, social services and legal support. These community responses support women experiencing violence by helping them access available resources and by engaging in community discussions. However, assistance is largely only offered to married women and responses tend to focus exclusively on physical rather than psychological or emotional forms of violence. Drawing on Campbell and Burgess’s (2012) framework for ‘community mental health competence’, we interrogate the potential implications of these responses for the mental wellbeing of women affected by violence. We conclude by drawing attention to the gendered nature of community responses to IPV and the potential impacts this may have for the mental health of women experiencing IPV.

## Introduction

How do communities respond to intimate partner violence (IPV) in resource-poor settings? We respond to this question by drawing on a case study of community responses to IPV in Rwanda and discussing its implications for women’s mental health. We take a broad understanding of mental health in this paper: one that encompasses the often severe mental disorders that can result from IPV experiences, but also pays attention to the ways in which controlling behaviours, psychological and emotional violence can undermine women’s mental wellbeing [1,2], and capacity to respond effectively to IPV. While IPV affects multiple groups including heterosexual men and women, transgender populations and those in same sex relationships [3], we have selected a focus on women in heterosexual relationships as the largest population affected by IPV globally [4] and for the lessons that can be learned for gendered relationships and acts of violence more broadly.

The World Health Organisation (WHO) defines IPV as ‘behaviour within an intimate relationship that causes physical, sexual or psychological harm, including acts of physical aggression, sexual coercion, psychological abuse and controlling behaviours’ [5]. Women who experience IPV have a heightened risk of severe mental disorders, including depression [6], anxiety [7], and post-traumatic stress disorder [8]. The absence of good mental health can significantly undermine the ability for women to respond to IPV by feeding a cycle of violence and disempowerment [9,10]. This contributes to particularly vulnerable situations for women in social environments where violence against women is socially accepted, often referred to as *coercive* settings for the ways in which women may blame themselves or their own behaviour for the violence in these settings [11–13]. The mental health effects of IPV are also compounded for women from stigmatised groups, such as LGBT, sex workers or migrants, whose experiences of violence are often not appropriately recognised by personal support networks or health providers [14–17]. Given complex interactions between experiences of IPV and the social setting in which women find themselves, interventions that focus solely on the psychiatric dimensions of the mental health consequences of IPV where women's mental wellbeing is addressed in isolation from the wider contextual realities are of limited value [1].

Recently, scholars have explored the potential for place-based communities or neighbourhoods to mediate the harmful impacts of IPV on women’s mental health in light of overwhelming evidence that social interaction leads to positive health outcomes [18–20]. When disclosing violence, women who receive positive and supportive responses from community members are less likely to experience post-traumatic stress [21]. Community members are also more likely to intervene in instances of IPV (e.g. by breaking up disputes or providing shelter) if they witness other members of their community intervening [22], and are more likely to take personal responsibility if IPV is perceived to be a community problem [23]. However, literature from resource-poor settings also highlights the challenges place-based communities present for addressing IPV and its mental health outcomes. Communities often respond with ambivalence to women experiencing violence and reinforce IPV as a normal part of women’s lives in ways that can be highly stigmatising [24,25]. Closely integrated communities can also deter women from disclosing violence for fear this will increase the severity of the violence from their partners [26]. Our own work in this area has pointed to the importance of supportive social environments (i.e. police, social services and government policy that support IPV prevention and response) in reversing community norms that condone violence and enabling communities to respond effectively to IPV-related behaviours [27].

While community-focused IPV interventions have shown some promising results, there is a dearth of evidence from resource-poor settings on the broader implications of these programmes. Community-based advocacy programmes have successfully assisted women in obtaining desired public resources, increased their overall sense of well-being, and reduced further risk of abuse [28]. However, these resource-intensive programmes are poorly suited for low-income countries in part because of a heavy reliance on well-established public services, including formal shelter systems. Bystander interventions and community mobilisation have brought about changes in knowledge and attitudes towards IPV [29–31], and community mobilisation interventions in particular have been highlighted as a promising approach for reducing physical and sexual IPV in resource-poor community settings [32]. However, these studies have focused exclusively on changes in attitudes, social norms and prevalence of violence with little attention paid to the broader implications for the community dynamics that drive IPV behaviours and/or fail to support women experiencing violence in the first place. Better understandings are needed.

We attempt to fill this gap by presenting a case study of a public policy from Rwanda that has attempted to engage place-based communities in responding to IPV by implementing gender-based violence (GBV) Committees in every village across the country (over 14,000 in total). We use this case study to first explore the types of responses communities use to address IPV and then consider the implications these responses may have for women’s mental health. In considering the potential implications of community IPV responses, we draw on a conceptual framework for ‘community mental health competence’ [33,34]. Campbell and Burgess [33] emphasise three key aspects of communities who are competent in addressing mental health. First, communities need *knowledge* to recognise signs and symptoms of distress and the services that are available. Second, communities require *safe social spaces* for thinking critically about the social and cultural drivers of poor mental health (including IPV), and how obstacles can be constructively overcome. Third, communities need *partnerships* with those who have the political power and the economic resources needed to effectively improve mental health outcomes. These three aspects of community mental health competence provide a useful framework for considering the enablers and barriers of community responses to IPV in mitigating women’s mental health in the discussion of our Rwandan case study.

### Rwanda’s national gender-based violence policy

In 2008, a Gender Based Violence Bill [35] was passed by the Rwandan parliament, which places an emphasis on the obligation of all Rwandan citizens to report violence: ‘Any person must prevent gender based violence, rescue and call for rescue the victims of this violence’ (p.94). Relevant punishments are outlined as part of the Bill, with ‘harassing one’s spouse’ and ‘conjugal rape’ levying punishments of imprisonment for 8 months to 2 years (p.97). Following the GBV Bill, a National Policy against Gender-Based Violence was developed by the Ministry of Gender and Family Promotion (MIGEPROF) in 2009. The policy provides support for women survivors within communities through establishing community-level (referred to locally as *umudugudu)* GBV Committees. Officially, six community members are elected to be part of the Committee, including: (1) the *umudugudu* Chief, (2) person in charge of social affairs, (3) the Representative of Women, (4) the person in charge of security, (5) a man and a woman selected for their exemplary integrity in the community, (6) the person in charge of information. The role of these Committees is to raise awareness about gender-based violence, identify and refer victims to appropriate services, report perpetrators to appropriate authorities, conduct home visits when gender-based violence is suspected, and report statistics on gender-based violence to other levels [36].

As a public policy, the GBV committees are fully integrated with Rwanda’s decentralised system of government. GBV Committees exist as each of the four levels of government: *umudugudu* (village), cell, sector and district. Gender-based violence cases that cannot be resolved at one level are moved up to the next highest level, which provides a chain of responsibility for addressing each individual case of GBV that is reported.

Based on 2015 figures from Rwanda, 33.6% of women have experienced physical violence from their current or most recent husband/partner [37]. Part and parcel of these prevalence rates are social norms that condone violence as a normal part of intimate relationships: 41.4% of Rwandan women and 17.9% of men believe that violent behaviour by a husband towards his wife is justified under specific circumstances [37].

## Methods

The data presented comes from a study of community responses to IPV in two Rwandan villages. Ethical approval for the study was granted by the London School of Economics and Political Science.

### Data collection

Data were collected over two different time periods in 2013 and in 2014. During the first period of data collection in 2013, the first author conducted four focus groups with six women each living in Kigali (n = 24). During the second period in 2014, the second author conducted two focus groups (one with men and one with women) and six interviews in each of two communities in Kigali (n = 35). The purpose of the project to understand how communities were responding to IPV in Rwanda was clearly described to each participant and the community leaders. All potential participants were engaged in a conversation about the project details, what we hoped to understand from the conversation (no personal stories or details were requested at any point during data collection), and the ability of participants to stop the interviews or focus groups at any time without giving a reason. These conversations between participants and the researchers lasted until all participants said they were happy to sign a consent form. In adherence to WHO guidelines for conducting research on domestic violence [38], the research team developed a comprehensive list of suitable supports available to women experiencing violence for referral in case participants disclosed personal experiences of violence. The ethical measures in place were adapted over the course of the project in order to engage with issues as they arose in a context-relevant way, as described elsewhere [39].

All interviews and focus groups covered similar topics: the meaning of IPV, community responses to IPV before and after the implementation of the GBV committees; referral mechanisms for difficult cases; and the level of support and training communities received from higher levels of government. The research team also discussed with participants the hypothetical case study of a woman named Claudine who lived in the village and was being beaten by her husband. Participants were asked to talk about their initial responses to this scenario. Once they had provided an initial response, additional information about Claudine was provided such as ‘Claudine’s husband rapes her’ or ‘Claudine has a child’, and participants were asked if anything about their response to the situation changed with this new information. This iterative approach to using a hypothetical case study provided detailed information for analysis about the factors contributing to community-level responses to women experiencing violence. Interviews and focus groups were recorded and transcribed into English for analysis.

### Participants

The participants in the study were all living in Kigali, the capital of Rwanda. A local research assistant selected participants for the first round of data collection drawing on her local contacts, church group and community. Two communities were selected for the second round of data collection, using pre-defined *umudugudu* boundaries. The local research assistants (one man and one woman) each made contact with the *umudugudu* Chief in the communities where they lived, and these Chiefs then recruited the rest of the research participants and provided a venue for the interviews. Within each *umudugudu*, six members of the GBV Committee were interviewed (see the explanation of GBV Committee roles above). Details of these participants and the corresponding method of data collection are summarised in Table 1.

**Table 1:** Participant details.

PARTICIPANTS	*N*	METHOD	SELECTION/ RECRUITMENT TECHNIQUE
**Round 1 data collection**			
Mixed community members (4 groups of 6 women)	24	Focus group discussions	Purposively selected by local research assistant
**Round 2 data collection**			
Members of GBV Committee (6 members, 2 communities)	12	In-depth interviews	Communities were purposively selected, Kigali-based
Community members (men)	12	Focus group discussion	Recruited through *umudugudu* leader
Community members (women)	11	Focus group discussion	Recruited through *umudugudu* leader
**TOTAL**	**59**		

### Data analysis

The transcribed interviews were entered into *Atlas*.*ti* for analysis. The first author read through 25% of the transcripts and created a preliminary coding frame for how communities were responding to IPV, with particular attention to how the response engaged or did not engage with the surrounding social environment in supporting women’s mental health. Community responses were identified and given preliminary codes according to which actors were responsible for responding to women experiencing violence (i.e. GBV committee, neighbours, local leaders, police). These ‘actor’ categories provided an initial means of organising the data, which was then analysed further to understand the different types of responses being undertaken by these different actors (referred to as overarching or ‘global’ themes following Attride-Stirling’s approach to thematic network analysis [40]). The second author used this coding frame to code the remaining transcripts and to validate or refine the coding frame as needed. Once this preliminary deductive coding was complete, the first author undertook a second inductive coding to identify basic in-vivo themes (summarising specific community activities in response to cases of IPV) drawing on participants’ own statements from the interviews and focus groups. The organising and global themes were then reorganised to reflect these in-vivo basic themes.

Conducting research on IPV as foreign researchers is fraught with ethical challenges related to the sensitive nature of the topic, and the ways in which our own subjectivities interact with the research context and may in turn influence our interpretation of the data. We have written at length about these considerations elsewhere, including the interest of participants in the researchers’ personal lives and details, and the challenge of obtaining consent for recorded interviews in a context where there is significant government surveillance over the private lives of individuals [39].

## Findings

Responses to IPV by community members both within and outside of GBV Committees’ responsibilities were categorised into three global themes: (1) Providing interpersonal support; (2) Engaging community support; and (3) Navigating public resources. These three themes were sequential and hierarchical: in most cases, communities would first try to reconcile couples.

### Providing interpersonal support: Couples counselling

Reconciling couples was the first response of most community members to IPV. This response focused on addressing interpersonal causes of violence between men and women, which were identified by participants as alcohol use, infidelity, and disagreements between couples. The GBV committee was said to play a major role in reconciling couples through trying to resolve these interpersonal arguments:

There are some who are really brutal, but here in our *umudugudu* we try to unify them and the man promises to no longer beat his wife. [Community A, Head of GBV Committee, Interviewed 02/05/2014]

The GBV committee also played an observational role, checking up on reports of violence and ensuring that these conversations with couples had indeed stopped the violence:

Interviewer: I want to ask about IPV specifically. What happens in your *umudugudu* when a married woman is beaten by her husband?

Participant: We call her to the GBV committee. We meet as the umudugudu committee with the person in charge of GBV. We call the man and the woman, we try to teach them, and we tell to the man that it is a crime to beat his wife. And then after that, we tell the committee to do a follow up after 2 days to check that the man is no longer beating his wife. [Community B, *Umudugudu* Chief, Interviewed 16/05/2014]

However, involvement of community members in providing interpersonal support also went beyond the GBV Committees. Elders, community leaders and neighbours were all mentioned as trying to stop IPV, often by reporting it to the authorities, and/or speaking directly to the couple:

Interviewer: What if you hear fighting and arguments from your neighbours. Would you be able to go and help them as an individual?

Participant: It happened once to my neighbour. She was pregnant and would be giving birth soon. Her husband became abusive and attacked her but she would not say anything…So, I went and told the authority and they came and intervened.

Interviewer: Was the husband angry at you for intervening?

Participant: I first talked to the wife and told her that she could not keep quiet about something like this and warned them that I would go to the authority.

[Community B, Focus group with women, Dated 18/05/2014]

This points to the high level of engagement of community members in cases of IPV. While the GBV committee is the formal body for intervention at the community level, neighbours and community elders also intervene in violent situations.

In providing interpersonal counselling-type support to couples, one of the main strategies used by the GBV committee to encourage reconciliation was advising the couple to marry if they were not already married. The rationale for this was two-fold: firstly, it was seen as a means of calming the conflict and thus reducing the violence:

Interviewer: Do you think that it is good to tell a woman who is abused, when she abused by her partner, to marry him?

Participant: Well, you first tell them about the best of living in harmony, but if they succeed to be legally married it is an advantage, she is protected from his unkindness, from him telling her to go away, from being treated like a prostitute because she is his wife. [Community A, Representative of Social Affairs, Interviewed 07/05/2014]

Secondly, encouraging unmarried couples to marry was seen as a means of ensuring that the rights of women and any children were protected. Encouraging the couple to separate was a last resort, only to be implemented if the violence was too severe:

The first thing is to advise them legalise their relationship. If we find that they are in conflict, it depends on the level of the conflict. If the conflict is high, we advise them to be separated before things become dangerous. [Community A, *Umudugudu* Chief, Interviewed 02/05/2014]

The level of severity of the violence played a significant role in the decisions made by community members about how to intervene in situations of IPV. As the Chief of Community A said: *“there is what we call beating and hurting*, *or beating her without hurting her”*. In situations where the violence was not perceived to be too severe, the best solution was almost always seen as trying to keep the couple together through counselling, and marriage was thought to provide an important foundation for a healthy relationship through improving the woman’s status in the relationship.

### Engaging community support

When attempts to reconcile the couple failed, community members often used public spaces as a means of putting pressure on men to change violent behaviour. There were two main public spaces that provided this opportunity. The first is a monthly meeting of the community, *umuganda*, mandated by the government to engage the community in ‘improvement’ projects, discuss local issues of concern and to hear from government authorities at higher levels. Bringing IPV cases to *umuganda* was seen as a means of encouraging public intervention and providing witnesses. The role of the *umuganda* was to assign an appropriate intervention team that would then follow up on the situation and report back once it was resolved. The use of *umuganda* for resolving cases of violence was contested among the community members we spoke to. Some saw it as a positive opportunity for open discussion and resolution of the case by other community members:

Participant: This is also what we talk about in the *umuganda*. When we know about a situation in the community that needs attention and follow up, this is where we speak about it.

Interviewer: You say their names in the *umuganda*?

Participant: Absolutely!

Participant: We might even go straight to their house.

Interviewer: Is this not confidential? Is it alright to do so publically?

Participant: No, it is fine. It has to be done so that everyone can see this, because you can be affecting the neighbourhood and causing disturbance in the whole community.

Participant: When you are abusing your wife, you are also affecting the whole community. [Community A, Focus group with men, Dated 09/05/2014]

However, others saw IPV as a private issue that did not have place in a public space such as *umuganda*:

Interviewer: Is it better when it goes in front of the *umuganda* meetings?

Participant: No it is not good.

Interviewer: Why?

Participant: Because the conjugal relationship is confidential. It is not good for everyone to know it. [Community A, Health worker, Interviewed 09/05/2014]

A second public space used as a space for public discussion of cases of IPV is *umugoroba w’ababyeyi*, or parents’ evenings. These community-level events are mandated by the Government’s GBV strategy. In the communities where we conducted this study, they occurred on an as-needed or regular basis and the attendees were primarily women (men were included in the events as ‘parents’, but often did not attend). Unlike the *umuganda*, *umugoroba w’ababyeyi* are not necessarily spaces for intervention, but rather an opportunity to tell one’s story and to ask for advice from other women in one’s community:

Participant: There was an example of a mother who had children, and also had an alcoholic husband. She came and shared with us in the evening for parents her concerns but what I can tell you is that both the evening for parents and the community leaders intervened and to this day, the family is doing much better.

[Community B, Focus group with women, Dated 18/05/2014]

The *umugoroba w’ababyeyi* plays a much less formal role than the *umuganda*. It is a space for listening and offering advice, whereas the *umuganda* provides a space for the resolution of community issues. However, both spaces provide public accountability for cases of violence–either member of a couple can bring concerns to these public spaces and have their stories heard and discussed.

### Navigating public resources

Calling on authority figures and public institutions, such as local police and the court system, was discussed as the last resort in situations of violence. Participants discussed two main pathways for engaging public resources in attempting to address IPV: (1) reporting the violence to the police, (2) reporting to higher levels of authority within government structures. The description of these two pathways highlights the limited public resources available to many women experiencing violence.

Police are called when the GBV committee is unable to appropriately counsel the couple or if the violence is considered severe (i.e. a knife is used). Police have the authority to place perpetrators of violence in jail, however, imprisoning perpetrators was described by participants as having multiple purposes. Perpetrators are jailed to punish individuals for GBV, separate violent couples for an unspecified period of time, and to scare individuals into changing their behaviour. The following except from a focus group with six men in one community highlights these multiple purposes:

Participant: One time I went to a police station where a husband had injured his wife, cutting her with a knife…So the wife comes early in the morning with bandages, she is injured; the police ask her what she is doing, what she wants. She replies that, she came to see her husband, mentions his name and the police asks her if she is referring to the man who injured his wife. She replies that she is the wife and that she wishes for them to let go of her husband so he could come home. So, the police ask the wife, “You want us to let go of a man who has hurt another human being, who injured you this much? Why did he hurt you in the first place? Was it you who started the fight or him? She replies that she started the fight…

Participant: The police took the wife and put her in jail also. [*Laughter*]

Interviewer: The police put her in jail?

Participant: Only to scare her. They did it to scare her saying that she started the fight, and she was involved in something bad. So, they let go of the husband instead, again to see their reaction. When the husband was let out of jail, he also refused to leave his wife behind. So, of course they had to let them both go.

[Community A, Focus group with men, Dated 09/05/2014]

This excerpt also sheds light on several limitations of imprisonment as a means of assisting women experiencing violence in this context. Gender inequalities are deeply embedded in this scenario, which contributed in this case to the victim being blamed for causing the violence and being jailed herself as punishment. These gender inequalities are rarely challenged since decisions about appropriate sentencing for perpetrators is allocated to the community whenever possible, giving police considerable decision-making power about who should be imprisoned, for how long, and with which types of evidence. The government’s preference for GBV cases to be resolved at the ‘location of the crime’ is written into the 2008 law on the Prevention on Punishment of Gender-based Violence (Chapter 3, Section 1, Article 12). Equally there are no stipulations in the law for punishing the perpetration of physical violence (although sexual violence, emotional violence, and adultery all have allocated sentences) [35].

When cases are deemed particularly complex or severe, the secondary pathway for engaging public resources is for the GBV Committee to report the case to the GBV Committee at cell level (a collection of *umudugudu*), and if that is not sufficient then to district level. In this way, each case of IPV that cannot be resolved through couples counselling or police intervention in the *umudugudu* is progressed up a chain of authority to higher and higher levels, with the final level being legal intervention by a court of law. This signposting can provide an important means of obtaining financial assistance for women experiencing violence. It also provided a means of addressing IPV, but only in cases where divorce was thought necessary:

When it reaches that level, when we see that there is deep violence, as she said, we take her away. We don’t have the right to do so; but we directly send her to the other level. In the past, we had such a case, and we brought our decision to the Cell, and they stamped it and she went to court. In that case a divorce was needed, before a crime occurred. It was to prevent a crime before it happened because when a couple fights like that there is a risk one of them will kill the other. [Community A, Head of GBV Committee, Interviewed 02/05/2014]

Although GBV is illegal in Rwanda, the role of the court is rarely to exact punishment for the criminal act of violence against women (although cases of GBV against children are often acted upon), but rather to separate the couple and divide their assets. As such, participants rarely saw the purpose of involving the court system for unmarried women experiencing violence. In these cases, decisions about the division of assets and custody of any children were arbitrated by community leaders. Therefore, while the law and government policies support women who are married and experiencing severe physical violence to separate from their husband, unmarried women and women experiencing forms of violence that may be less physically dangerous have little support.

## Discussion

Our findings on responses to IPV in two Rwandan communities highlight the active role community members including the GBV Committee, the *umudugudu* Chief, respected elders and neighbours play in responding to violence. This provides an encouraging contradiction to evidence from other resource-limited contexts of the ambivalence of communities to women experiencing IPV (Snell-Rood et al. 2015). Community members in Rwanda are not only listening to women and providing social support but engaging in public community forums as a means of putting pressure on men to stop the abuse and to ensure public accountability. Given evidence of the stress-reducing effects of supportive community responses for women who disclose violence (Edwards et al. 2015), this type of public community engagement may be enabling for these women. Community support in these two Rwandan communities also extended beyond these community-based spaces to obtain resources from higher levels of government including financial support and assistance for legal divorce. Financial support for women experiencing violence from intimate partners in low-income countries is well recognised as essential given evidence that many women in these contexts are dependent on their partners for their daily survival [41,42]. However, as mentioned in the findings, the responses by communities in Rwanda also raises important limitations around the kinds of women (i.e. married) and the kinds of violence (i.e. severe physical abuse) that are perceived as legitimately requiring government involvement at higher levels.

These results highlight some of the gendered nuances of community responses to IPV, but what are the mental health implications of different forms of response? Asking women about the implications of community responses for their mental health and wellbeing is an important direction for future research and a limitation of the current study. However, in considering the potential implications of community responses for women’s mental health, Campbell and Burgess (2012) provide a useful framework for assessing the mental health competencies of communities, which we use to benchmark our findings.

Campbell and Burgess (2012) first highlight the need for communities to have knowledge of the signs and symptoms of mental health distress and of appropriate and available services. Community responses to violence that include reconciling couples, encouraging women to marry violent partners, and that prioritise physical over emotional forms of violence all point to a lack of knowledge among community members of the damaging mental health impacts of IPV. Our findings show that community members were hesitant to recommend the separation of couples unless the violence was so severe that the woman’s life was clearly in danger. This is in spite of evidence suggesting that Rwandans do acknowledge psychological and emotional forms of violence as components of gender-based violence when asked what violence means to them [27]. This suggests that while Rwandan communities may have knowledge of IPV as including psychological as well as physical acts of violence, this may not be sufficient in changing how community members respond. This prioritisation of physical acts in responses to IPV should not be seen as limited to Rwandan communities however; legal scholars from the U.S. have long argued for attention to the psychological and emotional harms as part of state intervention in IPV cases [43,44].

The absence of a community response to psychological or emotional forms of violence is consistent with Campbell and Burgess’s [33] suggestion that while knowledge is necessary it is insufficient on its own. They suggest the need for social spaces that allow for unfamiliar knowledge to be ‘shared and debated’ with local frames of reference (p. 12). Applying this to our case study highlights the need for the evidence of causal links between IPV and women’s mental health to be shared and debated within community spaces. Our findings show that while community spaces do exist in *umuganda* and *umugoroba w’ababyeyi*, the extent to which these public forums foster open discussions about women’s mental health and the damaging effects of IPV requires further investigation. On the one hand, our findings point to how community forums are often used as spaces for reconciling couples in ways that may reinforce the hierarchy between the physical and psychological impacts of IPV mentioned previously. On the other hand, community psychologists have long emphasised the inherent value of constructive dialogue within communities and between communities and researchers as an end in and of itself [45,46]. Dialogue is seen as the foundation for critical thinking, or the process of ‘conscientisation’, whereby communities begin to see themselves as knowledgeable agents able to bring about change in their surrounding social and structural environment [47]. In our case study, the value of dialogue is evident in the opportunity that community spaces have provided for IPV to be openly discussed by community members, which inherently challenges the views of individuals who believe IPV should remain part of the private domain. In this way, these public spaces resemble what Arendt [48] describes as ‘spaces of appearance’–a public domain where people come together to discuss matters of common concern and to take action. According to Arendt, spaces of appearance are not spaces for building consensus, but rather are constantly disrupted by competing viewpoints; a space where participants are able to express themselves and realise their role as citizens. However, in relation to mental health, while these public spaces offer some protection for women experiencing violence by providing a supportive space for disclosure [21], they may also be limited in terms of what community members perceive to be legitimate forms of violence or who they perceive to be appropriate ‘victims’. In this way, the public spaces that are created do not go far enough in creating a space where emotional consequences and complex ideas around victimhood can be challenged in a way that lead to positive mental wellbeing among women affected by IPV.

The third component of Campbell and Burgess’ [33] framework for community mental health competence is partnerships between local communities and organisations or government agencies that have the ‘will and resources to support good mental health in the community’ (p. 390). Our findings point to a significant gap in mental health support for women experiencing IPV from police and government agencies. The decentralised government structure does provide mechanisms for supporting women experiencing IPV, including imprisoning perpetrators and accessing publically available funds. However, these support mechanisms are often undermined by gender norms as shown in the example of the woman imprisoned by police for admitting that she had started the disagreement with her husband. This action by police not only reaffirms gender norms that blame women for violence, but also compromises the forms of community solidarity and support that contribute to positive mental health outcomes (see [18,20]). Similarly, public funds for women experiencing IPV are only made available to certain women experiencing certain types of violence, which again is in opposition to supportive community structures for women’s mental health. The limited resources available to support the mental health of women experiencing IPV in Rwanda is consistent with a lack of resources for mental health in the country more broadly. While mental health has been a national priority for the Rwandan government, historically this priority has been significantly underfunded by international donors [49].

This analysis of the Rwandan case study through the lens of the community mental health competence framework points to the need for further research and a fully integrated approach to mental health and IPV intervention. There are some promising foundations for the potential of communities to respond to IPV in ways that could support women’s mental health at each level of the framework. Community members acknowledge psychological and emotional forms of violence, community forums are in place for public discussions about IPV and for planning interventions, and legal and financial mechanisms exist to support women experiencing severe physical IPV. However, our analysis also points to several remaining challenges: community responses to IPV often ignore the potentially damaging mental health impacts of violence, and community spaces as well as police and government services may reaffirm a prioritisation of the physical over psychological harm caused by IPV. In order to address these concerns, a more comprehensive approach to IPV intervention is likely needed; one that upholds the mental health implications of IPV as equal in importance to its physical impacts and puts in place supports for communities to respond effectively to all women experiencing IPV.

### Limitations

The data presented in this paper is from the capital city of Kigali, and the actions of GBV committees are likely different from other regions and more rural communities. The presence of female foreign researchers during the focus groups and interviews may also have influenced the types of responses that were given about the effectiveness and importance of GBV committee activities. However, this draws even more attention to the gaps identified in community responses to the implications of IPV for women’s mental health. In addition, another limitation is the use of the local chief to recruit participants during community discussions, and the ways this may have changed the demographics of the participants who agreed to take part. This was not a concern for the interviews with GBV Committee members since these roles are prescribed, but may have played a role in the focus group discussions with men and women in our two selected villages.

## Conclusions

The findings presented in this paper point to the need for further exploration of the implications of community-focused interventions to address IPV for women’s mental health. Rwanda provides a particularly interesting context for understanding community responses to IPV because of the local GBV committees mandated by policy. Outside of the Rwandan context, community mobilisation interventions to prevent IPV currently being implemented across Africa, Asia and Latin America [50] provide an enormous opportunity to investigate the implications for women’s mental health.

Our findings act as an important starting point for this further work. We have highlighted the potential for place-based communities to respond effectively to women experiencing IPV. This is an important and promising finding given the tendency for literature from resource-poor settings to emphasise the ‘harmful’ social norms implicated in community responses to IPV [51]. However, it is also clear that not all community responses are promising and that gender inequalities often act as a driver for community responses that are less supportive of women and their mental health needs.

## Supporting information

S1 Table Coding framework. (DOCX)

## References

[1] Burgess R, Campbell C. Contextualising women’s mental distress and coping strategies in the time of AIDS: A rural South African case study. Transcultural Psychiatry. 2014;51: 875–903. doi: 10.1177/1363461514526925 24670517 10.1177/1363461514526925

[2] Ryff CD, Keyes CLM. The structure of psychological well-being revisited. Journal of Personality and Social Psychology. 1995;69: 719–727. doi: 10.1037/0022-3514.69.4.719 7473027 10.1037//0022-3514.69.4.719

[3] Chen PH, Jacobs A, Rovi SL. Intimate partner violence: IPV in the LGBT community. FP essentials. 2013;412: 28–35. 24053263

[4] Devries KM, Mak JYT, García-Moreno C, Petzold M, Child JC, Falder G, et al The global prevalence of intimate partner violence against women. Science. 2013;340: 1527–1528. doi: 10.1126/science.1240937 23788730 10.1126/science.1240937

[5] Butchart A, Garcia-Moreno C, Mikton C, World Health Organization, London School of Hygiene and Tropical Medicine. Preventing intimate partner and sexual violence against women global trends and determinants of prevalence, safety, and acceptability [Internet]. Geneva: World Health Organization; 2010 Available: http://whqlibdoc.who.int/publications/2010/9789241564007_eng.pdf

[6] Kader Maideen SF, Sidik SM, Rampal L, Mukhtar F. Prevalence, associated factors and predictors of depression among adults in the community of Selangor, Malaysia. PloS one. 2014;9: e95395 doi: 10.1371/journal.pone.0095395 24755607 10.1371/journal.pone.0095395 PMC3995972

[7] Kader Maideen SF, Mohd Sidik S, Rampal L, Mukhtar F. Prevalence, associated factors and predictors of anxiety: a community survey in Selangor, Malaysia. BMC psychiatry. 2015;15: 262 doi: 10.1186/s12888-015-0648-x 26497745 10.1186/s12888-015-0648-x PMC4620008

[8] Kastello JC, Jacobsen KH, Gaffney KF, Kodadek MP, Bullock LC, Sharps PW. Posttraumatic stress disorder among low-income women exposed to perinatal intimate partner violence: Posttraumatic stress disorder among women exposed to partner violence. Archives of Women’s Mental Health. 2016;19: 521–528. doi: 10.1007/s00737-015-0594-0 26714487 10.1007/s00737-015-0594-0

[9] Dutton DG. The Abusive Personality, Second Edition: Violence and Control in Intimate Relationships. Guilford Press; 2006.

[10] Orava TA, McLeod PJ, Sharpe D. Perceptions of control, depressive symptomatology, and self-esteem of women in transition from abusive relationships. J Fam Viol. 1996;11: 167–186. doi: 10.1007/BF02336668

[11] Campbell C, Mannell J. Conceptualising the agency of highly marginalised women: Intimate partner violence in extreme settings. Global Public Health. 2016;11: 1–16. doi: 10.1080/17441692.2015.1109694 26669895 10.1080/17441692.2015.1109694

[12] Madhok S, Phillips A, Wilson K. Gender, agency and coercion. Basingstoke: Palgrave Macmillan; 2013.

[13] Mannell J, Jackson S, Umutoni A. Women’s responses to intimate partner violence in Rwanda: Rethinking agency in constrained social contexts. Global Public Health. 2016;11: 65–81. doi: 10.1080/17441692.2015.1013050 25734771 10.1080/17441692.2015.1013050

[14] Samudzi Z, Burgess R. "They don’t look at you as a person”: redefining mental health needs of Trangender sex workers in South Africa. (in preparation);

[15] Schumacher EC, Bishop CN, Capezza NM. Prejudice, discrimination and mental health status within the lesbian, gay and bisexual community. Psychology of Prejudice: New Research. 2014 pp. 121–134. Available: https://www.scopus.com/inward/record.uri?eid=2-s2.0-84954315844&partnerID=40&md5=f423a4dd595b5e3c6c950c52da41be72

[16] Shahmanesh M, Wayal S, Cowan F, Mabey D, Copas A, Patel V. Suicidal behavior among female sex workers in Goa, India: The silent epidemic. American Journal of Public Health. 2009;99: 1239–1246. doi: 10.2105/AJPH.2008.149930 19443819 10.2105/AJPH.2008.149930 PMC2696657

[17] Yoshihama M. Reinterpreting strength and safety in a socio-cultural context: Dynamics of domestic violence and experiences of women of Japanese descent. Children and Youth Services Review. 2000;22: 207–229. doi: 10.1016/S0190-7409(00)00076-1

[18] Coker AL, Smith PH, Thompson MP, McKeown RE, Bethea L, Davis KE. Social Support Protects against the Negative Effects of Partner Violence on Mental Health. Journal of Women’s Health & Gender-Based Medicine. 2002;11: 465–476. doi: 10.1089/15246090260137644 12165164 10.1089/15246090260137644

[19] Kamimura A, Parekh A, Olson LM. Health Indicators, Social Support, and Intimate Partner Violence Among Women Utilizing Services at a Community Organization. Women’s Health Issues. 2013;23: e179–e185. doi: 10.1016/j.whi.2013.02.003 23660431 10.1016/j.whi.2013.02.003

[20] Wright EM, Pinchevsky GM, Benson ML, Radatz DL. Intimate Partner Violence and Subsequent Depression: Examining the Roles of Neighborhood Supportive Mechanisms. American Journal of Community Psychology. 2015;56: 342–356. doi: 10.1007/s10464-015-9753-8 26391793 10.1007/s10464-015-9753-8

[21] Edwards KM, Dardis CM, Sylaska KM, Gidycz CA. Informal Social Reactions to College Women’s Disclosure of Intimate Partner Violence: Associations With Psychological and Relational Variables. Journal of Interpersonal Violence. 2015;30: 25–44. doi: 10.1177/0886260514532524 24811285 10.1177/0886260514532524

[22] Palmer JE. Recognizing the continuum of opportunities for third parties to prevent and respond to sexual assault and dating violence on a college campus. Crime Prevention and Community Safety. 2016;18: 1–18. doi: 10.1057/cpcs.2015.18

[23] Beeble ML, Post LA, Bybee D, Sullivan CM. Factors related to willingness to help survivors of intimate partner violence. Journal of Interpersonal Violence. 2008;23: 1713–1729. doi: 10.1177/0886260508314333 18319363 10.1177/0886260508314333

[24] Odero M, Hatcher AM, Bryant C, Onono M, Romito P, Bukusi EA, et al Responses to and Resources for Intimate Partner Violence: Qualitative Findings From Women, Men, and Service Providers in Rural Kenya. Journal of Interpersonal Violence. 2014;29: 783–805. doi: 10.1177/0886260513505706 24255067 10.1177/0886260513505706 PMC3910289

[25] Snell-Rood C. Informal support for women and intimate partner violence: the crucial yet ambivalent role of neighbours in urban India. Culture, Health & Sexuality. 2015;17: 63–77. doi: 10.1080/13691058.2014.950333 25204832 10.1080/13691058.2014.950333

[26] Naved RT, Azim S, Bhuiya A, Persson LÅ. Physical violence by husbands: Magnitude, disclosure and help-seeking behavior of women in Bangladesh. Social Science & Medicine. 2006;62: 2917–2929. doi: 10.1016/j.socscimed.2005.12.001 16426717 10.1016/j.socscimed.2005.12.001

[27] Mannell J, Dadswell A. Preventing Intimate Partner Violence: Towards a Framework for Supporting Effective Community Mobilisation. J Community Appl Soc Psychol. 2017;27: 196–211. doi: 10.1002/casp.2297

[28] Bybee DI, Sullivan CM. The process through which an advocacy intervention resulted in positive change for battered women over time. American journal of community psychology. 2002;30: 103–32. doi: 10.1023/A:1014376202459 11928772 10.1023/A:1014376202459

[29] Abramsky T, Devries KM, Michau L, Nakuti J, Musuya T, Kyegombe N, et al The impact of SASA!, a community mobilisation intervention, on women’s experiences of intimate partner violence: secondary findings from a cluster randomised trial in Kampala, Uganda. J Epidemiol Community Health. 2016; jech-2015-206665. doi: 10.1136/jech-2015-206665 26873948 10.1136/jech-2015-206665 PMC4975800

[30] Hines DA, Palm Reed KM. Predicting Improvement After a Bystander Program for the Prevention of Sexual and Dating Violence. Health Promotion Practice. 2015;16: 550–559. doi: 10.1177/1524839914557031 25380846 10.1177/1524839914557031

[31] Wagman JA, Gray RH, Campbell JC, Thoma M, Ndyanabo A, Ssekasanvu J, et al Effectiveness of an integrated intimate partner violence and HIV prevention intervention in Rakai, Uganda: analysis of an intervention in an existing cluster randomised cohort. The Lancet Global Health. 2015;3: e23–e33. doi: 10.1016/S2214-109X(14)70344-4 25539966 10.1016/S2214-109X(14)70344-4 PMC4370228

[32] Ellsberg M, Arango DJ, Morton M, Gennari F, Kiplesund S, Contreras M, et al Prevention of violence against women and girls: what does the evidence say? The Lancet. 2015;385: 1555–1566. 10.1016/S0140-6736(14)61703-7 25467575

[33] Campbell C, Burgess R. The role of communities in advancing the goals of the Movement for Global Mental Health. Transcultural Psychiatry. 2012;49: 379–395. doi: 10.1177/1363461512454643 23008350 10.1177/1363461512454643

[34] Burgess R, Mathias K. Community Mental Health Competencies: A New Vision for Global Mental Health In: White RG, Jain S, Orr DMR, Read UM, editors. The Palgrave Handbook of Sociocultural Perspectives on Global Mental Health. London: Palgrave Macmillan UK; 2017 pp. 211–235. doi: 10.1057/978-1-137-39510-8_11

[35] Rwanda Law No. 59/2008. Prevention and Punishment of Gender-Based Violence [Internet]. Oct 9, 2008 p. 26. Available: http://www.refworld.org/docid/4a3f88812.html

[36] MIGEPROF. Guidelines for setting up GBV committees [Internet]. Kigali, Rwanda: Ministry of Gender and Family Promotion; 2009 7 Available: www.migeprof.gov.rw%2FIMG%2Fdoc%2FGUIDELINES_FOR_SETTING_UP_GBV_COMMITTEES.doc&ei=RuqTVaXqK4uAU-Sel8gI&usg=AFQjCNHqg9mzZVFQEZdYYMzmhyFjiLTYGA&sig2=isxDx8sasJ8xwOf1mk2azQ&bvm=bv.96952980,d.d24&cad=rja

[37] National Institute of Statistics Rwanda. Demographic and Health Survey 2014/2015—Key findings [Internet]. Kigali, Rwanda: Ministry of Finance and Economic Planning; 2016 Available: http://www.statistics.gov.rw/publication/demographic-and-health-survey-dhs-20142015-key-findings

[38] World Health Organisation. Putting women first: Ethical and safety recommendations for research on domestic violence against women [Internet]. Geneva, Switzerland: Department of Gender and Women’s Health; 2001 1 p. 32. Available: http://www.who.int/gender-equity-rights/knowledge/who_fch_gwh_01.1/en/

[39] Mannell J, Guta A. The ethics of researching intimate partner violence in global health: A case study from global health research. Global Public Health. 2017;0: 1–15. doi: 10.1080/17441692.2017.1293126 28278750 10.1080/17441692.2017.1293126

[40] Attride-Stirling J. Thematic networks: an analytic tool for qualitative research. Qualitative Research. 2001;1: 385–405.

[41] Rogers B. The Domestication of Women: Discrimination in Developing Societies. London: Kogan Page; 1980.

[42] Susser I. AIDS, Sex, and Culture: Global Politics and Survival in Southern Africa. Wiley-Blackwell; 2009.

[43] Johnson ME. Redefining Harm, Reimagining Remedies, and Reclaiming Domestic Violence Law. UC Davis L Rev. 2008;42: 1107.

[44] Mills LG. Killing Her Softly: Intimate Abuse and the Violence of State Intervention. Harvard Law Review. 1999;113: 550–613. doi: 10.2307/1342332

[45] Campbell C, Jovchelovitch S. Health, community and development: towards a social psychology of participation. Journal of Community & Applied Social Psychology. 2000;10: 255–270. doi: 10.1002/1099-1298(200007/08)10:4<255::AID-CASP582>3.0.CO;2-M

[46] Jovchelovitch S. Knowledge in Context: Representations, Community and Culture. London: Routledge; 2007.

[47] Freire P. Pedagogy of the Oppressed: 30th Anniversary Edition Bloomsbury Publishing; 2000.

[48] Arendt H. The Human Condition. 2nd ed (1998). Chicago: University of Chicago Press; 1958.

[49] Saraceno B, Ommeren M van, Batniji R, Cohen A, Gureje O, Mahoney J, et al Barriers to improvement of mental health services in low-income and middle-income countries. The Lancet. 2007;370: 1164–1174. doi: 10.1016/S0140-6736(07)61263-X 10.1016/S0140-6736(07)61263-X 17804061

[50] Michau L, Horn J, Bank A, Dutt M, Zimmerman C. Prevention of violence against women and girls: lessons from practice. The Lancet. 2015;385: 1672–1684. doi: 10.1016/S0140-6736(14)61797-9 10.1016/S0140-6736(14)61797-9 25467577

[51] VanderEnde KE, Yount KM, Dynes MM, Sibley LM. Community-level correlates of intimate partner violence against women globally: A systematic review. Social Science & Medicine. 2012;75: 1143–1155. doi: 10.1016/j.socscimed.2012.05.027 22762950 10.1016/j.socscimed.2012.05.027

