# Supplementary material for: The implications of community responses to intimate partner violence in Rwanda
Source: PLoS One. 2018 May 2;13(5):e0196584. doi: 10.1371/journal.pone.0196584 (PMC5931791; doi:10.1371/journal.pone.0196584)
Supplement: S1 Table — (DOCX) [file pone.0196584.s001.docx]

**Coding framework: Community responses to intimate partner violence**

| **ORGANISING THEME** | **BASIC THEME** | **IN-VIVO TEXT** |
| --- | --- | --- |
| **Global theme 1: Providing interpersonal support: Couples counselling** | | |
| GBV committees | Advise the couple to legalise the relationship (get married)  Help couples separate if the violence is severe  Ask men who abuse their wives to join the committee  Analyse the conflict in order to attribute a cause or blame  Offer advice on alternatives to violence  Tell men about the negatives of IPV  Distribute household resources to women and children  Report severe cases to the police or the cell GBV committee | ‘The first thing [the committee does] is advise them to legalize their relationship.’  ‘If [the GBV committee] judge that their case complicated, we ask the *umudugudu* to separate them, especially when it is dangerous for one of them.’  ‘He accused his wife of sleeping with other young men and was following her everywhere. [The GBV committee] talked with him and told him that this was not good.’  ‘Together with another committee member we were able to approach him and counsel him and though at first he insisted that he provided enough for his family, he began to realize his faults and agreed to change.’ |
| Neighbours | Neighbours are encouraged to report violence  Neighbours have an obligation to report IPV (legal and ethical)  Neighbours are called on to provide support  Neighbours give advice to other community members experiencing IPV | ‘We ask to the people to give information about any problem that happens between families.’  ‘When you hear of violence and you don’t participate in stopping it, it may become worse.’  ‘When it is a woman [who reports violence] we send another woman to go speak to her and helping her solve the problem.’  ‘The umudugudu may send you to a family that has the same problem.’ |
| Community advisors | Advice and counselling is provided by local women  Elders in the community provide advice | ‘As an elder woman, women often come to me to seek advice and counsel.’  ‘We have an elder woman who is in charge of fighting against GBV.’ |
| Local leaders | Leaders decide how property should be divided in cases of separation  Leaders try to reconcile couples who are fighting  Men trust leaders | ‘We have great leaders who really have intervened and tried all their best to be mediators, visiting these homes and reconciling them.’ |
| **Global Theme 2: Engaging community support** | | |
| Umuganda | A space for changing men’s behaviour through public confrontation  A space for training/ providing information  A space where members of the GBV committee are voted for | ‘Most of the cases spoken about in umuganda are cases that are not secret, the whole community knows and is talking about it. These are cases where the man has been abusive many times and he has been spoken to but nothing changes.’  ‘In umuganda, the community leaders talk about GBV... GBV is an important issue to talk about because it affect homes. So, they have to talk about it on a regular basis.’ |
| Parent’s evenings (umugoroba w’ababyeyi) | A space for sharing family concerns  A woman’s space for talking about violence and receiving advice  A space for intervention | ‘There was an example of a mother who had children and an alcoholic husband. She came and shared with us her concerns in the evening for parents.’ |
| **Global theme 3: Navigating public resources** | | |
| Court system | Courts arbitrate the division of property in cases of divorce  Courts take decisions on child support payments  Courts take decisions on custody of children in the case of married couples  No legal recourse is possible if the couple is not married | ‘The man is ordered by the court to pay child support until the children is 7 years old.’  ‘If a woman is not married and goes to the court to claim anything, there is no evidence or proof to support her and all they can tell is that she has to be married.’ |
| Local police | Severe cases are referred to the police  Police use station ‘jails’ to resolve violence | ‘[In cases of violence,] if we find that the problem is beyond our capacity we send it to the police.’  ‘When the man attacks with a knife or when rape happens, these cases go to the police.’ |
